# A Weight Loss Intervention for African American Breast Cancer Survivors, 2006

**Published:** 2008-12-15

**Authors:** Melinda R. Stolley, Lisa K. Sharp, April Oh, Linda Schiffer

**Affiliations:** University of Illinois at Chicago; University of Illinois at Chicago; University of Illinois at Chicago; University of Illinois at Chicago

## Abstract

**Introduction:**

Breast cancer survival rates are lower for African American women than for white women. Obesity, high-fat diets, and lack of regular physical activity increase risk for breast cancer recurrence, comorbid conditions, and premature death. Eighty-two percent of African American women are overweight or obese, partly because of unhealthy eating and exercise patterns. Although successful weight loss and lifestyle interventions for breast cancer survivors are documented, none has considered the needs of African American breast cancer survivors. This study assessed the feasibility and impact of Moving Forward, a culturally tailored weight loss program for African American breast cancer survivors.

**Methods:**

The study used a pre-post design with a convenience sample of 23 African American breast cancer survivors. The 6-month intervention was theory-based and incorporated qualitative data from focus groups with the targeted community, urban African American breast cancer survivors. Data on weight, body mass index (BMI), diet, physical activity, social support, and quality of life were collected at baseline and at 6 months.

**Results:**

After the intervention, we noted significant differences in weight, BMI, dietary fat intake, vegetable consumption, vigorous physical activity, and social support.

**Conclusion:**

This is the first published report of Moving Forward, a weight loss intervention designed for African American breast cancer survivors. Although a randomized trial is needed to establish efficacy, the positive results of this intervention suggest that this weight loss intervention may be feasible for African American breast cancer survivors. Lifestyle interventions may reduce the disparities in breast cancer mortality rates.

## Introduction

Breast cancer is the second leading cause of cancer death among African American women (1). Despite lower incidence rates, mortality is higher for African American women than for white women (1). The ethnic disparities in Chicago, Illinois, exceed national rates; the mortality rate is 68% higher for African American women than for white women (2). Several factors may account for the ethnic differences in breast cancer survival rates, including later stage of presentation, lack of insurance, and other health care barriers (3). Furthermore, African American breast cancer survivors are more likely to die from comorbid conditions than are their white counterparts (4). Obesity and lifestyle behaviors are 2 contributors to higher mortality rates that are worthy of attention because they may be modifiable.

The 2003-2004 National Health and Nutrition Examination Survey (NHANES) showed that approximately 82% of African American women are overweight or obese (body mass index [BMI] ≥25.0 kg/m^2^), compared with 58% of white women (5). Moreover, studies suggest that African American women gain more weight than do white women after a breast cancer diagnosis (6). This weight gain may be partially attributed to lifestyle variables, including increased caloric consumption and decreased physical activity (6). Data for African American women in the general population show unhealthy eating patterns and low rates of regular physical activity (7,8). Obesity, high-fat diets, and low levels of physical activity are associated with shorter survival and increased risk of recurrence in both premenopausal and postmenopausal women (9,10).

Healthy eating and exercise should be promoted among breast cancer survivors for many reasons. Such behaviors can lead to weight loss or weight gain prevention, which may translate into reduced risk for cancer recurrence (11,12) and comorbid conditions (4), along with improved quality of life (13). These benefits may be even greater for African American survivors, given their higher risk for hypertension, diabetes, and heart disease (14). Furthermore, weight gain causes distress and compromises quality of life for African American breast cancer survivors (15,16).

In recent years, a few weight loss interventions have been developed for breast cancer survivors (17-20). All report beneficial results, including decreases in weight (17,19,21), prevention of weight gain (20), improved body composition (19,22), improved blood lipid levels (19), decreased dietary fat intake (20), increased physical activity (20) improved psychological status (20), and increased fruit, vegetable, or fiber intake (20). However, African American women were not well represented in these studies.

Because of the high breast cancer mortality and obesity rates among African Americans, African American breast cancer survivors should be encouraged to participate in weight loss interventions. However, data suggest that African American women are less likely to participate in traditional weight loss programs, are more likely to drop out, and lose less weight than do white women because of both biological and cultural factors (23,24). To meet the needs of African American breast cancer survivors, weight loss programs must be culturally sensitive and incorporate the practices, attitudes, and beliefs of this particular group (25). Results of recent qualitative studies of weight loss among African American women generally (25), and breast cancer survivors specifically (15,16), share common themes. Overall, African American women prefer programs that provide holistic and practical information on improving diet and physical activity patterns and that consider barriers and facilitators to weight loss. Barriers include family and social obligations, poor social support, financial limitations, and limited access to physical activity and healthy eating resources. Facilitators include religious faith, social support, and the awareness that they are taking steps to decrease risk for cancer recurrence and comorbid conditions (16). Weight loss efforts are also influenced by such factors as taste and the role of food within the African American culture (15,16,25). These unique cultural contributors to weight loss in the African American community were considered integral in the development of Moving Forward, a comprehensive weight loss intervention designed for urban African American breast cancer survivors. This article presents feasibility and impact data of Moving Forward.

## Methods

### Recruitment

Full institutional review board approval was obtained from the University of Illinois at Chicago, where the study was conducted. Recruitment was conducted in collaboration with the local chapters of 2 national breast cancer support organizations, Sisters Network, Inc, and Y-ME National Breast Cancer Organization. Both provide information and social support to women diagnosed with breast cancer. Y-ME (currently known as Breast Cancer Network of Strength) serves a diverse clientele, whereas Sisters Network, Inc, focuses on the needs of African American women. A total of 100 women who were current or past participants in support or educational programs at Y-ME and Sisters Network, Inc, received information on the program. Of these, 38 expressed interest in the program and 23 were eligible. Eligibility requirements included 1) being at least 18 years old, 2) self-identifying as black/African American, 3) having a stage I, II, or III breast cancer diagnosis, 4) having a BMI ≥25 kg/m^2^, 5) having completed breast cancer treatment (except endocrine treatment) at least 6 months before baseline interview, 6) having physician approval to participate in a moderate physical activity program, 7) not using prescription weight loss medications, 8) not currently participating in organized weight loss programs, and 9) being willing and able to complete the preintervention and postintervention interviews and attend twice weekly classes for 6 months. Reasons for ineligibility included inability to find transportation to attend the program and conflict with work hours.

### Procedure

Participants completed interviews at baseline and postintervention. The interview required 60 to 90 minutes and included questions on demographics and breast cancer diagnosis and treatment, as well as measures of height and weight, dietary and physical activity patterns, social support for eating and exercise, and quality of life.

### Measures

All measures except the Satisfaction Questionnaire were administered by the interviewer. Interviews were conducted by 2 people with extensive training and experience in collecting health behavior and psychosocial data. Interviewers received training on each questionnaire and on how to measure height and weight, conducted mock interviews, and were certified by a senior, "master" interviewer. For quality control purposes, 2 interviews for each interviewer were taped and reviewed for consistency.

Height was measured by using a Seca 214 portable stadiometer (seca gmbh & co, Hamburg, Germany) and weight was measured by using a Tanita BWB-800 digital scale (Tanita Corporation of America, Inc, Arlington Heights, Illinois). Participants removed their shoes and any heavy outer clothing for the anthropometric measurements.

The Block '98 Food Frequency Questionnaire (FFQ) was developed from NHANES food intake data and includes a food list that was derived separately for African Americans, whites, and Hispanics. Reliability and validity were established for the measure in a wide range of age, sex, income, and ethnic groups (26). Data from the FFQ can be used to calculate nutrient intake, food group intake, and other dietary variables.

The International Physical Activity Scale, Long Format (long IPAQ) is designed to assess physical activity during the previous 7 days. Items assess physical activity across a diverse set of domains, including leisure time, domestic and yard, work-related, and transport-related physical activity. Participants are asked only to report activity that they engaged in for at least 10 minutes at a time. Separate scores are calculated for walking, moderate-intensity activity, and vigorous-intensity activity. The psychometric properties of the IPAQ compare favorably to other commonly used self-reported physical activity measures (27).

The Social Support for Eating and Exercise questionnaire asks respondents to rate on a 5-point scale (1 = never, 5 = very often) the frequency with which friends and family have done or said certain things related to the respondents' efforts to change dietary or exercise habits. The eating survey has 2 subscales each for friends and family — encouragement and discouragement, which can range from 5 to 25. Higher scores on the encouragement scale mean better social support; higher scores on the discouragement scale mean lower social support. Friend support for exercise has 1 subscale, participation, which can range from 10 to 50. Family support for exercise is made up of 2 subscales, participation and rewards and punishment. Because of low internal consistency (Cronbach α = 0.36), results are not reported for the rewards and punishment subscale. Higher scores on the participation scale mean better social support (28).

Quality of life was measured by the Functional Assessment of Cancer Therapy (FACT)-B (breast) and FACT-ES (endocrine symptoms). The FACT-B consists of the FACT-G (general) with 4 subscales: physical well-being, social/family well-being, functional well-being, and emotional well-being, plus a breast cancer subscale. Higher scores indicate a higher quality of life. Data reflect high internal consistency (α = 0.90) and good test-retest reliability for this measure (29). FACT-ES assesses the side effects and putative benefits of hormonal treatments for breast cancer. FACT-ES has good internal consistency and test-retest reliability (30).

The satisfaction measure was developed to gather information on satisfaction with the intervention and its specific components. The questionnaires were distributed on the last day of class, and women were asked to return them in an anonymous envelope within 2 weeks. Questionnaires were mailed to women who were not in attendance.

### Intervention

The intervention was developed by integrating concepts from 2 health behavior change theories, Social Cognitive Theory (31) and the Health Belief Model (32). Social Cognitive Theory suggests that personal change occurs as a result of the dynamic interaction between modifications in behavior, cognition (attitudes, knowledge, self-efficacy), and the environment (social support). Mechanisms by which change is encouraged include modeling and reinforcement. The Health Belief Model incorporates the concepts of perceived severity, susceptibility, benefits, and barriers (32). To initiate change, participants must experience their vulnerability to risk and recognize the benefits of initiating behavioral change. Content and structure of the intervention were organized to provide 1) information to increase knowledge and improve attitudes about diet, physical activity, and weight loss and their relationship to breast cancer prognosis and general health; 2) opportunity to enact positive behavioral changes and increase self-efficacy; 3) an environment in which participants felt comfortable in applying problem-solving skills, allowing them to confront barriers to change; and 4) reinforcement and social support for making health behavior changes.

In addition to Social Cognitive Theory and the Health Belief Model, the intervention also incorporated tenets related to the practice of culturally competent research (33). Culturally sensitive interventions require the recognition of the beliefs and practices of the particular social, ethnic, and age group for whom the intervention is being developed, appreciation of the roles these factors play in participants’ lives, and considerate incorporation into the intervention (33). On the basis of focus groups with African American breast cancer survivors (16) and information culled from the literature, we focused on food, family, music, social roles and relationships, and spirituality/religion. Tailored cultural considerations included 1) addressing the importance of food in the African American culture and ways to integrate this value with healthful eating, 2) providing low-fat versions of traditional soul food recipes, 3) incorporating a physical activity component that addressed barriers to regular physical activity (safety, weather, access, time), 4) acknowledging family roles and family resistance to change, 5) providing information on the value of healthful lifestyles for children and spouses, 6) facilitating social support for making changes in diet and physical activity, and 7) understanding the role of religion and worship in the lives of these women and how it affected their health perspectives.

The intervention took place over 6 months and included 2 weekly classes. The first weekly class was 2 hours and involved discussions of knowledge, attitudes, barriers, facilitators, benefits, and costs related to changes in diet, exercise, and weight (see Table 1 for weekly topics). These discussions often included hands-on activities such as weighing and measuring foods according to participants' typical portions and then according to recommended portions, a field trip to the grocery store to practice reading food labels, creating weekly meal plans, and preparing a healthier version of a particular dish. The last 60 minutes involved an exercise class. The second weekly class was also an exercise class. Exercise classes were taught by a certified exercise instructor who also conducted community-based classes in African American neighborhoods. She incorporated a variety of activities in her classes, including traditional aerobics, line dancing, African dance, salsa, yoga, Pilates, strength training, and flexibility training. Social support was a component of the program. Activities to promote group cohesion were incorporated into the intervention. Monday night classes began with an ice breaker that focused on participants' experiences as breast cancer survivors with topics such as "What was the most difficult phase of your breast cancer experience?" and "What is your funniest memory of your experience?" The group shared potluck dinners in honor of holidays, a breast cancer advocate who has done much for the African American community in Chicago, and significant events (1 woman's 5-year survival anniversary, the Moving Forward graduation). Occasionally, activities outside of the intervention were also planned. These included a cancer survivor walk, the American Cancer Society Making Strides walk, and Cancer Survivor Day at a professional baseball game. Friends and family were invited to all classes and activities.

### Statistical analyses

Descriptive statistics for demographic variables and for baseline BMI and weight were computed for all participants. Only participants with postintervention data were included in the attendance and other analyses. In most cases paired *t* tests were used to evaluate whether the mean change in an outcome variable was significantly different from zero. However, because the physical activity data were not normally distributed, Wilcoxon signed-rank tests were used to determine whether the median change in physical activity was significantly different from zero. The median is recommended as the preferred measure of central tendency for the IPAQ (27). SAS for Windows version 9.1 (SAS Institute, Inc, Cary, North Carolina) was used for all analyses.

## Results

Twenty-three women were eligible to participate in the program, and 20 completed both the preintervention and postintervention interviews. Three women withdrew after the start of the intervention, but none attended any classes. Two of these women stated that they were no longer interested in participating, and the third was unable to find reliable transportation. Despite the small sample, the group included a diverse range of ages (30.6 to 70.1 years), education (eighth grade or less through graduate or professional degree), and annual income (less than $10,000 to more than $75,000) (Table 2).

Attendance and satisfaction data support the acceptability and feasibility of the program. Forty-six classes were offered. The average number of classes attended was 31 (SD 12). Nearly 55% of the 20 participants attended at least 75% of the classes. Satisfaction questionnaires were completed by 90% of the women. These results reflected little variance; all respondents reported that they enjoyed all parts of the program in the format they were presented. The most common responses to the item, "List the most important things you gained from the program" were social support, information related to food labels and portions, a sense of empowerment to make lifestyle changes, and the opportunity to exercise in a structured and supportive environment.

Participants experienced significant changes in both weight and BMI during the intervention (Table 3). For the 20 women with postintervention data, mean weight loss was 5.6 pounds. The decrease in BMI was 1.0 kg/m^2^.

Baseline FFQ data revealed diets high in fat and low in fruit and vegetable consumption (Table 3). Vegetable consumption increased significantly after the intervention (1.6 servings per day), as did fiber grams per 1,000 kcal (3.8 g per 1,000 kcal). Total daily fat consumption decreased significantly (23.6 g), though the decrease in the percentage of energy from fat (3.4%) was not significant. Total daily energy intake decreased, though not significantly (377.0 kcal). The changes in sodium consumption and in servings of fruit per day were not significant.

Median time spent in vigorous activity increased significantly during the intervention, from 0 minutes per day at baseline to 23.6 minutes per day (Table 4). Although time spent in moderate activity and in all physical activity also increased, these changes were not significant.

Baseline scores on the social support subscales reflected low levels of support for healthy eating and exercise patterns (Table 5). In general, social support improved significantly between the baseline and postintervention interviews. However, discouragement by friends also increased significantly.

Participants' baseline total FACT-G scores were high, with a mean of 88.2 of a possible 108, as were scores for each of the 4 subscales. Moving Forward participants reported better quality of life than did the normative sample of women in the general population used for the FACT-G. As for breast cancer symptoms and endocrine symptoms, the women reported few problems and high quality of life, higher than the normative sample of breast cancer patients used for the FACT-B and FACT-ES (30). No significant changes in quality of life were found over the course of the study.

## Discussion

This article is the first published report of a weight loss intervention designed for African American breast cancer survivors. The success of Moving Forward is most likely due in part to the involvement of African American breast cancer survivors in developing the intervention. Pilot results support the feasibility of recruiting, enrolling, and maintaining African American breast cancer survivors in a 6-month weight loss intervention. Our retention rate was 87%, and more than half of the participants attended 75% of all sessions, reflecting a high level of motivation. These recruitment and retention rates are similar to those seen in weight loss interventions among white breast cancer survivors (17,20,22) and higher than those seen for African American women participating in general weight loss programs (34,35).

Participants in Moving Forward exhibited significant weight loss, improved diet, increases in vigorous physical activity, and increased social support related to healthy eating and exercise. The mean weight loss for participants was 5.6 pounds (SD 6.5 pounds) or 3% of initial body weight (SD 3.7%) in 6 months. Similar interventions with white survivors have reported weight loss ranging from 3.6 to 13.2 pounds (9,17,20,21). A 5.6-pound weight loss is within the range of weight loss (0 to 9.9 lb) noted for interventions with healthy African American women (24,34,35). Only 3 (15%) of the participants lost 7% or more of their starting weight, a goal set by the investigators on the basis of what is considered clinically significant by the National Heart, Lung, and Blood Institute. However, most women were pleased with their achievements. Many explained that weight loss was a goal, but equally important goals were making a commitment to the program and making incremental changes in their lifestyles. This highlights an inherent, albeit small, conflict between what the investigators hoped for and what the participants actually strove for. Whereas weight loss was a common goal, the amount of desired weight loss differed for the 2 parties. A lesson in this pilot, and likely for many intervention studies, is achieving a balance between the investigators' and the participants' interpretations of success. Although 1 woman was clearly motivated by weight loss, losing a total of 16 pounds, other participants measured success in ways that had more personal meaning. For example, 1 participant with a long history of depression felt success for having nearly perfect attendance in the program, and another participant believed that consistently exercising 3 times per week was more important than weight loss. Regardless of these differences in goals, most participants "graduated" from Moving Forward reporting that they felt empowered to live a more healthful lifestyle.

Overall, participants made a number of positive dietary and physical activity changes that could lower their risk for breast cancer recurrence and other comorbid conditions (4,11). Dietary changes included a reduction in sweet fatty foods such as desserts and increased vegetable consumption, which brought their total daily fruit and vegetable consumption (7.6 servings, 2.1 fruits, 5.5 vegetables) to above the recommended 7 per day (36). Although modest changes in dietary fat were noted in this study, future interventions for breast cancer survivors should consider encouraging deeper reductions. The Women's Intervention Nutrition Study showed that reducing dietary fat intake to approximately 20% of total energy significantly reduced participants' risk for breast cancer recurrence (11).

Although the increase in moderate physical activity was not significant, median time spent in vigorous activity increased from 0 to 24 minutes. This increase was most likely a direct result of the intervention, which offered 2 weekly exercise classes (with 20 to 30 minutes of vigorous activity) and exercise DVDs that the women could use at home. Several women were able to integrate regular physical activity into their lives outside of class, but for many this remained a challenge. Most worked full-time, and many served as caregivers to children, elderly parents, or grandchildren, a common situation for many African American women. As a result, time, energy, and motivation were the 3 primary barriers. In discussions on ways to overcome these barriers, the most accepted solution was attending the Moving Forward classes. This is of concern because the long-term benefits of exercise require ongoing participation in physical activity, and these women may have stopped exercising after Moving Forward ended. Given the importance of exercise for weight loss and its independent contribution to improved breast cancer prognosis (10), it may be helpful for future interventions to focus on ways to sustain physical activity by facilitating women's participation in community sources of physical activity.

Finally, social support is a component of successful weight loss (37). At the start of Moving Forward, women reported low levels of family and friend support for both healthy eating and exercise. However, significant increases in social support were noted over time. Intervention activities were organized to include the participants' friends and family members and to encourage high group cohesion. Health disparity research highlights the interdependence of culture and psychosocial issues and the significance of kinship networks for support in making personal health decisions in the African American community (38). Negative social support via friend discouragement also increased significantly as participants realized that their friends outside the group were not particularly supportive of their efforts to make healthy eating and exercise choices. Meals at friends' houses and at church were often high-fat and unhealthy. The positive social support of the group probably contributed to the high level of participation and retention.

Despite improvements in weight, diet, exercise, and social support, no differences were noted in quality of life over the course of the intervention. This finding may reflect a ceiling effect, as women's quality of life scores were high at baseline (mean FACT-G score of 88.2). Comparisons of FACT-G scores to normative samples of both African Americans in general (79.6) and African Americans with cancer (78.5) show that scores were higher for Moving Forward women. Scores on the FACT-B and FACT-ES subscales were also higher for Moving Forward women.

Several limitations deserve consideration when interpreting the results of this study. The sample size was small, and there was no comparison group. Subject recruitment was based on self-selection and thus may have resulted in a biased sample. Measures of dietary intake and physical activity were based on self-report, a method that lends itself to recall bias. Social desirability may also have influenced reporting of dietary and physical activity patterns. Finally, these results cannot be generalized to other populations. To truly understand the efficacy of the intervention, larger translational research in this area is needed.

Results from this study support the feasibility of a weight loss intervention for African American breast cancer survivors. The intervention was created on the basis of information culled from the literature and a series of focus groups with African American breast cancer survivors. Outcome results include significant weight loss, improvements in diet and physical activity patterns, and increased social support relating to healthy eating and exercise. Numerous reports address the need to achieve and maintain a healthy weight and adopt healthy eating and exercise patterns to enhance breast cancer survival (4,10-12,39). Few interventions, however, address the specific needs or wants of African American women. Such interventions may reduce the disparities currently seen in breast cancer mortality rates.

## Figures and Tables

**Table 1:** Weekly Curriculum Topics, Moving Forward Weight Loss Intervention for African American Breast Cancer Survivors, 2006

Week	Topic
1	Obesity, lifestyle behaviors and breast cancer — an overview
2	Pros and cons of weight loss, tools for weight loss, self-monitoring
3	Tools for weight loss: water, fruit and vegetable intake, planning for Thanksgiving
4	Tools for weight loss: physical activity, goal setting
5	Portions and food labels
6	Healthy holiday strategies
7	Holiday party
8	Check-in: self-rating diet and physical activity patterns
9	Increasing physical activity in your daily life, pedometers
10	Meal planning
11	Healthy grocery shopping
12	Fast food, fast fat
13	Barriers to making healthy changes
14	Problem-solving techniques
15	Finding motivation, visit from long-term survivor and advocate
16	Check-in: self-rating diet and physical activity patterns
17	Emotional eating
18	Hidden calories
19	Review of personal barriers and facilitators
20	Exercise: finding opportunities at home, at work, in your community
21	Stimulus control
22	Social cues and eating/exercise patterns
23	Relapse prevention
24	Graduation celebration

**Table 2:** Participant Characteristics at Baseline (N = 23), Moving Forward Weight Loss Intervention for African American Breast Cancer Survivors, 2006

**Variable**	Mean (SD) or n
**Age, y**	51.4 (8.9)
**Education**	
High school graduate, GED, or less	3
Some college	10
College graduate	4
Graduate or professional degree	6
**Income, $ **	
<20,000^a^	3
20,000-24,999	2
25,000-34,999	2
35,000-49,999	5
50,000-74,999	8
≥75,000	3
**Employment status **	
Full-time	16
Unemployed	1
Retired	4
Disabled	2
**Marital status **	
Single, never married	4
Married or living with partner	8
Separated	1
Divorced	10
**Stage at diagnosis **	
I	8
II	11
III	1
Unknown	3
**BMI, kg/m^2^**	34.7 (7.8)
25 to <30	9
30 to <35	5
35 to <40	4
≥40	5
**Weight, lb**	197.9 (47.9)

Abbreviations: GED, General Educational Development certificate.

a The income categories <$10,000, $10,000-$14,999, and $15,000-$19,999 were merged.

**Table 3:** Weight, BMI, and Dietary Factors at Baseline and Postintervention, Women With Follow-Up Data Only (N = 20), Moving Forward Weight Loss Intervention for African American Breast Cancer Survivors, 2006

**Variable**	Baseline Mean (95% CI)	6 Months Mean (95% CI)	Mean Change (95% CI)	*P* Value^a^
Weight, lb	193.5 (174.6 to 212.4)	187.9 (168.8 to 207.0)	−5.57 (−8.63 to −2.51)	.001
BMI, kg/m^2^	34.1 (30.8 to 37.4)	33.1 (29.8 to 36.4)	−1.00 (−1.55 to −0.46)	.001
Energy, kcal/day	1,820 (1,454 to 2,187)	1,443 (1,142 to 1,745)	−377.0 (−795.3 to 41.2)	.07
Fat, g/day	78.6 (59.7 to 97.4)	55.0 (41.8 to 68.2)	−23.6 (−44.4 to −2.7)	.03
Fat, % kcal/day	38.1 (34.5 to 41.6)	34.6 (30.9 to 38.4)	−3.4 (−7.6 to 0.8)	.10
Fiber, g/day	17.8 (14.1 to 21.5)	19.4 (15.1 to 23.6)	1.6 (−2.1 to 5.2)	.38
Fiber, g/1,000 kcal	9.9 (8.6 to 11.3)	13.7 (11.3 to 16.1)	3.8 (1.3 to 6.3)	.005
Sodium, mg/1,000 kcal	1,307 (1,206 to 1,408)	1,404 (1,213 to 1,595)	96.7 (−91.5 to 284.9)	.30
Vegetables, servings/day	3.8 (2.7 to 5.0)	5.5 (3.7 to 7.2)	1.6 (0.0 to 3.2)	.05
Fruits, including juices, servings/day	1.7 (1.2 to 2.1)	2.1 (1.5 to 2.7)	0.5 (−0.2 to 1.1)	.16

Abbreviations: BMI, body mass index; CI, confidence interval.

a Paired *t* tests were used to determine significance.

**Table 4:** Physical Activity at Baseline and Postintervention, Women With Follow-Up Data Only (N = 20), Moving Forward Weight Loss Intervention for African American Breast Cancer Survivors, 2006

**Variable**	Baseline Median (IQR)	6 Months Median (IQR)	Median Change (IQR)	*P* Value^a^
Walking, min/day	28.6 (12.9 to 65.7)	26.4 (18.6 to 66.4)	3.9 (−32.9 to 23.6)	.78
Moderate activity, min/day	19.6 (7.5 to 62.5)	40.7 (23.6 to 85.0)	20.0 (−4.6 to 34.3)	.17
Vigorous activity, min/day	0 (0 to 11.1)	23.6 (7.5 to 44.6)	12.5 (1.4 to 43.6)	.02
Total physical activity, min/day	61.8 (22.1 to 198.2)	92.1 (78.9 to 212.1)	38.9 (−18.2 to 93.6)	.21

Abbreviation: IQR, interquartile range.

a Wilcoxon signed rank tests were used to determine significance.

**Table 5:** Social Support at Baseline and Postintervention, Women With Follow-Up Data Only (N = 20), Moving Forward Weight Loss Intervention for African American Breast Cancer Survivors, 2006

**Variable**	Baseline Mean (95% CI)	6 Months Mean (95% CI)	Mean Change(95% CI)	*P* Value^a^
**Healthy eating**				
Family encouragement	9.5 (6.7 to 12.3)	13.0 (9.7 to 16.3)	3.5 (1.2 to 5.8)	.005
Friend encouragement	8.5 (6.6 to 10.3)	13.6 (10.6 to 16.6)	5.2 (2.7 to 7.6)	<.001
Family discouragement	10.8 (8.5 to 13.0)	10.1 (8.0 to 12.1)	−0.7 (−2.4 to 1.0)	.39
Friend discouragement	10.1 (8.3 to 11.8)	12.4 (10.3 to 14.5)	2.4 (0.6 to 4.1)	.01
**Exercise**				
Family participation	18.4 (14.1 to 22.6)	22.6 (16.7 to 28.4)	4.2 (−0.1 to 8.5)	.06
Friend participation	18.3 (15.5 to 21.1)	23.4 (19.2 to 27.6)	5.1 (0.6 to 9.6)	.03

Abbreviation: CI, confidence interval.

a Paired *t* tests were used to determine significance.
